# The Efficacy of *Bifidobacterium longum* BORI and *Lactobacillus acidophilus* AD031 Probiotic Treatment in Infants with Rotavirus Infection

**DOI:** 10.3390/nu9080887

**Published:** 2017-08-16

**Authors:** Myeong Soo Park, Bin Kwon, Seockmo Ku, Geun Eog Ji

**Affiliations:** 1 Department of Hotel Culinary Arts, Yeonsung University, Anyang 430-749, Korea; mspark@yeonsung.ac.kr; 2 Research Center, BIFIDO Co. Ltd., Hongcheon 250-804, Korea; 531083@hanmail.net; 3 Fermentation Science Program, School of Agribusiness and Agriscience, College of Basic and Applied Sciences, Middle Tennessee State University, Murfreesboro, TN 37132, USA; Seockmo.Ku@mtsu.edu; 4 Department of Food and Nutrition, Research Institute of Human Ecology, Seoul National University, Seoul 151-742, Korea

**Keywords:** probiotics, rotavirus, *Bifidobacterium*, *Lactobacillus*

## Abstract

A total of 57 infants hospitalized with rotavirus disease were included in this study. The children were randomly divided into the study’s two treatment groups: three days of the oral administration of (i) a probiotics formula containing both *Bifidobacterium longum* BORI and *Lactobacillus acidophilus* AD031 (*N* = 28); or (ii) a placebo (probiotic-free skim milk, *N* = 29) and the standard therapy for diarrhea. There were no differences in age, sex, or blood characteristics between the two groups. When the 57 cases completed the protocol, the duration of the patients’ diarrhea was significantly shorter in the probiotics group (4.38 ± 1.29, *N* = 28) than the placebo group (5.61 ± 1.23, *N* = 29), with a *p*-value of 0.001. Symptoms such as duration of fever (*p* = 0.119), frequency of diarrhea (*p* = 0.119), and frequency of vomiting (*p* = 0.331) tended to be ameliorated by the probiotic treatment; however, differences were not statistically significant between the two groups. There were no serious, adverse events and no differences in the frequency of adverse events in both groups.

## 1. Introduction

Diarrhea-associated deaths in children under five years old in developing countries have been a major cause of childhood mortality [1]. These illnesses are caused by multiple factors, including infections by pathogenic microorganisms, viruses, and parasites [2]. Among the many acute diarrheal diseases, infections caused by rotavirus may be more fatal in infants than in adults [3]. Global reports show that most babies and toddlers are infected with rotavirus by the age of five [4]. This causes serious problems in developing and/or low-income countries (e.g., South Asian and sub-Saharan African countries), and hundreds of thousands of babies are killed by rotavirus annually [5]. Recently, the developments of rotavirus vaccines (e.g., RotaTeg and Rotarix) have dramatically reduced the number of outbreaks in many countries and were proven safe; however, concerns remain regarding the cost of the rotavirus vaccines and their limited effectiveness in some cases [6]. Accordingly, supported therapeutic methods that are compatible with common rotavirus medical treatments and effectively relieve its symptoms should be developed.

A number of studies have identified the effect of several probiotic species (e.g., *Bifidobacterium*, *Enterococcus*, *Lactobacillus*, *Lactococcus*, *Propionibacterium*, *Saccharomyces* and *Streptococcus*) in the treatment and prevention of intestinal infections [7]. These probiotic bacteria have been shown to inhibit intestinal disease [8,9,10,11]. *Bifidobacterium* and *Lactobacillus* spp. are the most common bacteria and are considered the most beneficial probiotic organisms [12].

Although multiple probiotic microorganisms could be utilized in rotavirus treatments, some studies have not identified any significant therapeutic effects; therefore, the underlying mechanisms of the therapeutic effects of probiotics in humans are still unclear [13]. Studies have shown that some probiotic bacteria have little or no statistically significant effect on rotavirus [14,15]. Moreover, we can deduce that the effect of probiotics may vary based on the type of microorganism administered to the host. We aim to determine the efficacy of a commercially available probiotic product containing two probiotic cell types, i.e., *Bifidobacterium longum* BORI and *Lactobacillus acidophilus* AD031, in infants and/or toddlers with rotavirus-associated symthoms.

## 2. Materials and Methods

Design: All participants’ guardians completed written, informed consent forms prior to the clinical experiment. All patients were recruited and classified from the inpatient Department of Pediatrics at Yonsei University Hospital in Seoul, Korea. This double-blind, randomized, and placebo-controlled clinical study tests the efficacy of probiotics formula to ameliorate the pathological symptoms in children hospitalized with rotavirus infections. The criteria applied to the experimental subjects are as follows: nine- to 16-month-old male and female infants were diagnosed as infected with rotavirus via a latex agglutination test. A total of 57 infants hospitalized with rotavirus infection were enrolled in this study. 28 patients were assigned to the probiotics treatment group, and the remaining 29 patients were assigned to the placebo group. The probiotics group was fed probiotic formula containing *B. longum* BORI and *L. acidophilus* AD031.

Diet and probiotic microorganisms: The probiotic powder contained two lyophilized probiotic species. Each probiotic packet contained 20 billion CFU/g of *B. longum* BORI and two billion CFU/g of *L. acidophilus* AD031 in powder form. The probiotics-free skim milk powder (placebo packet) was not visually distinguishable from the composite probiotic packet. Both the probiotic and placebo packets were supplied by BIFIDO Co., Ltd. (Hongchun, Korea). Each participant consumed the packets (i) twice a day (ii) for a total of three days (iii) within 10 min of each meal.

Statistical analysis: Paired *t*-tests were performed to assess the quantitative changes in the symptoms of rotavirus infection: duration of fever, frequency of diarrhea, frequency of vomiting, and duration of diarrhea before and after the study period in both groups. Results were considered statistically significant when the *p*-values were < 0.05.

## 3. Results and Discussion

A total of 57 infants hospitalized with rotavirus infection were enrolled in this study. Twenty-eight patients were randomly assigned to the probiotics group and 29 to the placebo group. The probiotics group was fed a probiotic formula containing *B. longum* BORI and *L. acidophilus* AD031. There were no differences in the age, sex, or blood characteristics of the two groups. The experimental outcomes are summarized in Table 1. The probiotics group showed a slightly reduced duration of fever (*p* = 0.119), frequency of diarrhea (*p* = 0.119), and frequency of vomiting (*p* = 0.331) compared to the placebo group; however, these differences were not significant. By contrast, the duration of diarrhea during the three-day treatment showed a significant difference between the probiotics group (4.38 ± 1.29) and the placebo group (5.61 ± 1.23) with a *p*-value of 0.001 (Table 1). There were no serious, adverse events and no difference in the frequency of adverse events in both groups.

The probiotic formula containing *B. longum* BORI and *L. acidophilus* AD031 utilized in this work is likely be an effective adjuvant to relieve acute diarrhea caused by rotavirus. Several studies showed that various strains of probiotic bacteria, such as *L. reuteri* and *L. rhamnosus*, were effective in managing acute diarrhea caused by rotavirus in toddlers. In the present experiment, the efficacy of *B. longum* BORI and *L. acidophilus* AD031 probiotic products was tested. Our rationale for the *L. acidophilus* and *B. longum* combination was based on the general microbial composition, which shows a predominance of *Lactobacillus* sp. in the small intestine and *Bifidobacterium* sp. in the large intestine (among a variety of beneficial bacteria present in healthy human subjects). Eighteen of 23 clinical trials of probiotic formulas resulted in mitigating acute diarrhea, and the reduction of the duration of diarrhea in the studies’ probiotics treatment group was reported to be 0.5 to 1.5 days [16]. The duration of diarrhea may vary depending on a child’s health status, diet, and prescribed medication. Our study demonstrated a statistically significant diarrhea reduction of 1.2 days. The efficacy of probiotics is strain-specific, so this may be due to the use of different strains in different studies. Basu et al. [17] conducted a clinical study with 10^7^ CFU/day LGG and concluded that it was not effective, but when they performed the same study again [18] with 10^10^ and 10^12^ CFU/day LGG, they concluded that a higher concentration of LGG administration in acute diarrhea patients was effective in reducing the diarrhea frequency, diarrhea period, and hospitalization period. Fang et al. [19] reported a dose-dependent effect of *Lb. rhamnosus* on fecal rotavirus concentration and suggested 6 × 10^8^ CFU/day as the minimal effective dose, which was similar to the data of Guanidalin [20], who concluded that at least 10 billion cells/day was necessary. Dubay [21] also applied the commercially available probiotic formula (VSL#3, CD Pharma India, New Delhi, India) to mitigate acute diarrhea, which showed a more rapid recovery compared to the control group and decreased the necessity of electrolyte treatments. In contrast to the positive results mentioned above, a probiotic formula containing 10^9^ CFU/day of *B. lactis* and 10^8^ CFU/day of *S. thermophilus* failed to decrease the duration of rotavirus diarrhea [22]. These contrasting results suggest that further clinical experiments are necessary in order to understand the scientific basis of the efficacy of probiotics and its relation to a number of criteria the strain of probiotics, the type of rotavirus, the severity of the symptoms, the ages and races of the children, etc. Further study using animal models also should be considered since the experimental conditions in this model can be better controlled [23,24,25,26,27]. 

## 4. Conclusions

The results of the present study demonstrated that a probiotic formula containing *Bifidobacterium longum* BORI and *Lactobacillus acidophilus* AD031 reduced the duration of rotavirus diarrhea in young Korean children.

## Figures and Tables

**Table 1:** Duration and frequency of rotavirus-associated symptoms in patients treated with probiotics and placebo.

Symptoms		Condition		*p-*Value
Category	Parameter	Placebo (*N* = 29)	Probiotics (*N* = 28)	
Duration ***(Days)***	Fever	4.32 ± 1.94	3.66 ± 1.14	0.119
	*Diarrhea*	*5.61 ± 1.23*	*4.38 ± 1.29*	*0.001*
Frequency (Times/Day)	Vomiting	1.82 ± 0.94	1.55 ± 1.12	0.119
	Diarrhea	2.64 ± 0.73	2.38 ± 0.49	0.331
